# Predicting occult lymph node metastasis in solid-predominantly invasive lung adenocarcinoma across multiple centers using radiomics-deep learning fusion model

**DOI:** 10.1186/s40644-024-00654-2

**Published:** 2024-01-12

**Authors:** Weiwei Tian, Qinqin Yan, Xinyu Huang, Rui Feng, Fei Shan, Daoying Geng, Zhiyong Zhang

**Affiliations:** 1 Academy for Engineering and Technology, Fudan University, No. 220 Handan Road, Shanghai, 200433 China; 2 Department of Radiology, Ruijin Hospital, Shanghai Jiao Tong University of Medicine, No. 197 Ruijin Er Road, Shanghai, 200025 China; 3 School of Computer Science, Shanghai Key Laboratory of Intelligent Information Processing, Fudan University, No. 2005 Songhu Road, Shanghai, 200433 China; 4 Department of Radiology, Shanghai Public Health Clinical Center, Fudan University, No. 2501 Caolang Road, Shanghai, 201508 China; 5 Department of Radiology, Zhongshan Hospital, Fudan University, No. 180 Fenglin Road, Shanghai, 200032 China; 6 Fudan University, No. 220 Handan Road, Shanghai, 200433 China

**Keywords:** Deep learning, Feature fusion, Occult lymph node metastasis, Radiomics, Solid-predominantly invasive lung adenocarcinoma

## Abstract

**Background:**

In solid-predominantly invasive lung adenocarcinoma (SPILAC), occult lymph node metastasis (OLNM) is pivotal for determining treatment strategies. This study seeks to develop and validate a fusion model combining radiomics and deep learning to predict OLNM preoperatively in SPILAC patients across multiple centers.

**Methods:**

In this study, 1325 cT1a-bN0M0 SPILAC patients from six hospitals were retrospectively analyzed and divided into pathological nodal positive (pN+) and negative (pN-) groups. Three predictive models for OLNM were developed: a radiomics model employing decision trees and support vector machines; a deep learning model using ResNet-18, ResNet-34, ResNet-50, DenseNet-121, and Swin Transformer, initialized randomly or pre-trained on large-scale medical data; and a fusion model integrating both approaches using addition and concatenation techniques. The model performance was evaluated by the area under the receiver operating characteristic (ROC) curve (AUC).

**Results:**

All patients were assigned to four groups: training set (*n* = 470), internal validation set (*n* = 202), and independent test set 1 (*n* = 227) and 2 (*n* = 426). Among the 1325 patients, 478 (36%) had OLNM (pN+). The fusion model, combining radiomics with pre-trained ResNet-18 features via concatenation, outperformed others with an average AUC (aAUC) of 0.754 across validation and test sets, compared to aAUCs of 0.715 for the radiomics model and 0.676 for the deep learning model.

**Conclusion:**

The radiomics-deep learning fusion model showed promising ability to generalize in predicting OLNM from CT scans, potentially aiding personalized treatment for SPILAC patients across multiple centers.

**Supplementary Information:**

The online version contains supplementary material available at 10.1186/s40644-024-00654-2.

## Background

Lung cancer remains the leading cause of cancer-related mortality globally [1]. Surgical resection is currently recognized as the primary treatment option for early-stage cases. In invasive lung adenocarcinoma, the solid predominant subtype serves as a substantial prognostic factor for both mortality and recurrence in patients undergoing resection. Additionally, it exhibits a strong correlation with a higher incidence of lymph node involvement [2–4]. Computed tomography (CT) scanning and positron emission tomography (PET)/CT scanning are the main non-invasive techniques for pre-surgical lymph node metastasis diagnosis. Yet, patients often progress from a clinical N0 (cN0) diagnosis to a pathological N1 (pN1) or N2 (pN2) stage after surgery, a condition termed occult lymph node metastasis (OLNM) [5].

The accurate preoperative identification of OLNM in solid-predominantly invasive lung adenocarcinoma (SPILAC), particularly for tumors with a solid component diameter of 2 cm or smaller, has emerged as a key focus and challenge for radiologists and thoracic surgeons. On the one hand, the OLNM status can assist in stratifying patients and providing therapeutic decision-making. High-risk patients may benefit from lobectomy and radical lymph node dissection, leading to improved survival, while low-risk patients may opt for less invasive procedures, such as sublobectomy or wedge resection, to enhance their quality of life [6, 7]. On the other hand, the interpretation of lymph node short-axis diameter on CT has been shown to be unreliable in diagnosing lymph node metastasis [8]. A meta-analysis revealed that PET/CT assessment for OLNM in non-small cell lung cancer patients can result in false positives due to inflammation and granuloma [9]. Additionally, the high cost associated with PET/CT poses a significant challenge to its broad clinical implementation [10, 11].

Recent studies [12, 13] have utilized CT-based radiomics for lymph node metastasis prediction. Radiomics features possess interpretability but are limited to extracting information solely from the tumor region, neglecting the relationship with surrounding tissues. Deep learning, particularly convolutional neural networks (CNNs) [14] and Transformers [15], has shown great potential in automatically capturing representative information from the entire image. A few works [16, 17] developed deep learning methods to predict lymph node metastasis for lung cancer. However, these studies had limited sample sizes or failed to account for lymph node characteristics. Furthermore, these models, developed and validated in single-center studies, faced challenges in broader clinical applications. Diverging from these approaches, our study: (1) amassed a substantial, multi-center dataset of SPILAC patients, emphasizing clinical generalizability; (2) integrated radiomics and deep learning, enhancing the predictive model’s performance; (3) explored various model architectures, data initialization methods, and feature fusion techniques to bolster generalization capabilities.

Thus, the purpose of this study was to develop and validate a radiomics-deep learning fusion model for OLNM prediction in SPILAC patients across multiple centers.

## Methods

### Patients and images

**Fig. 1:**
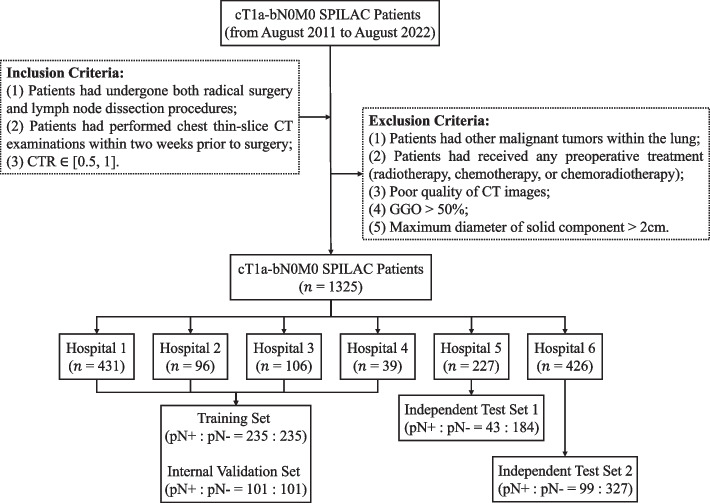
Flowchart illustrating the process of patient enrollment and the criteria for inclusion and exclusion in the dataset. CTR = consolidation-to-tumor ratio, GGO = ground-glass opacity, pN +  = pathological nodal positive, pN- = pathological nodal negative

This multi-center retrospective study was approved by the institutional review boards of the six hospitals (Additional file 1: Appendix A), and the requirement for obtaining informed consent from the patients was waived. A total of 1325 patients with cT1a-bN0M0 SPILAC who were admitted for treatment between August 2011 and August 2022 were selected. Figure 1 presents the inclusion and exclusion criteria for patient screening in this study. The training and internal validation sets from hospitals 1–4 were generated by randomly splitting patients with pN+ and pN- cases in a 7:3 ratio, respectively. To select pN- patients, a 1:1 matching ratio based on gender, age, density, and nodular location was employed with pN+ patients in the training and internal validation sets. As for the two independent test sets, all eligible patients in hospitals 5–6 with pN+ and pN- cases were included.

Preoperative consecutive thin-slice CT images of 1325 patients were obtained from the picture archiving and communication system (PACS). Among the 1325 CT images, 899 images were non-enhanced, while the remaining 426 images were contrast-enhanced. A total of 70 mL of contrast agent was intravenously injected with bolus at a flow rate of 3.5–4 mL/s. The contrast-enhanced scans were acquired at 30–35 s. The CT imaging protocols were detailed in Additional file 1: Appendix A.

### Image preprocessing

Thin-slice CT images (slice thickness ≤ 2 mm) of all patients were imported into the uAI Research Portal (uRP, Shanghai United Imaging Intelligence, Co., Ltd, China) in DICOM format for initial automatic tumor region segmentation. In the absence of knowledge regarding the pathological results, an experienced radiologist manually corrected the tumor masks slice-by-slice on the axial images obtained from the non-enhanced or contrast-enhanced phase in fixed lung window (window level: -600 HU, window width: 2000 HU), generating the three-dimensional volumes of interest (VOIs). Both the images and the masks were isotropically resampled to a voxel size of 1 × 1 × 1 mm^3^ using bilinear interpolation and nearest neighbor interpolation, respectively, for resolution normalization. The VOIs corresponding to the tumor regions served as the inputs for the radiomic model. Subsequently, we selected images of size 96 × 96 × 96 voxels containing the entire tumor regions by padding and cropping, which served as inputs to the deep learning model.

### Radiomics model development

The workflow for the development of the radiomics model includes (a) Feature extraction: A total of 1364 radiomics features were extracted from the delineated three-dimensional VOIs based on uRP. (b) Feature selection: Ten most important radiomics features were selected using a decision tree. (c) Model development: A radiomics-based model for predicting OLNM was constructed using the support vector machine. For detailed information on the radiomics approach, please refer to Additional file 1: Appendix B.

### Deep learning model development

To investigate the impact of model architecture and data initialization strategies on the performance of OLNM prediction in SPILAC across multiple centers, five common neural networks, namely ResNet-18 [18], ResNet-34 [18], ResNet-50 [18], DenseNet-121 [19], and Swin Transformer [20], were employed to build classification models separately. Beyond training from scratch, both ResNet and Swin Transformer models could be pre-trained on extensive medical datasets and subsequently fine-tuned on our specific dataset, aligning with the paradigm of transfer learning. The ResNet was pre-trained through segmentation tasks on the 3DSeg-8 dataset [21], which includes CT/magnetic resonance (MR) images of 1638 cases from different organs/tissues. The Swin Transformer was pre-trained on a set of 5050 CT cases derived from various organs. The pre-training was accomplished through three proxy tasks of self-supervised learning: masked volume inpainting, contrastive learning, and rotation prediction [22]. We developed a total of nine deep learning models. By feeding the output of each neural network into a global average pooling layer, we were able to obtain deep learning features.

### Radiomics-deep learning fusion model development

**Fig. 2:**
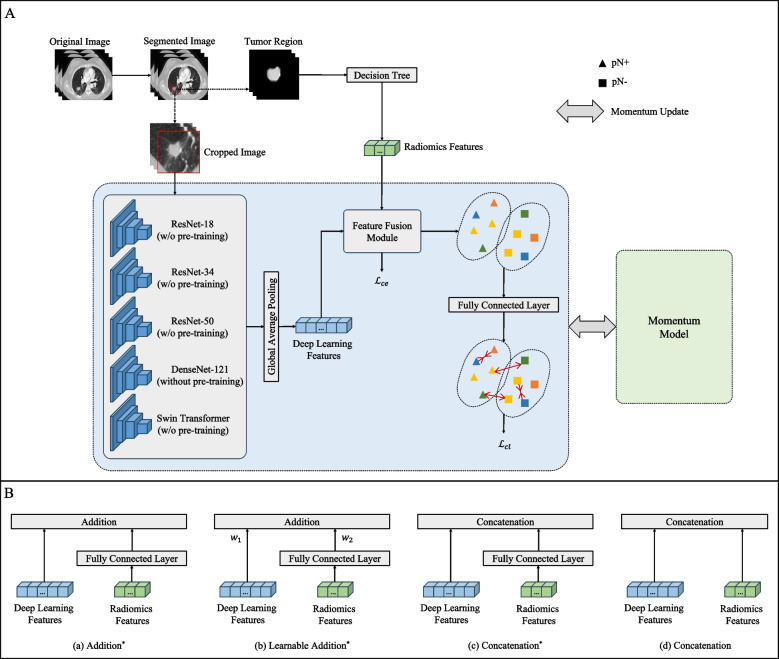
Design of the study. **A** Overall framework of the radiomics-deep learning fusion model for the prediction of OLNM in SPILAC. The w/o stands for with/without. ${\mathcal{L}}_{ce}$ and ${\mathcal{L}}_{cl}$ represent the cross-entropy loss function and the contrastive learning loss function, respectively. **B** Comparison of different feature fusion techniques. ^*^ denotes the mapping of radiomics features to the same dimension as the deep learning features through the fully connected layer. ${w}_{1}$ and ${w}_{2}$ represent the learnable weight parameters

The architecture of radiomics-deep learning fusion model was depicted as detailed in Fig. 2. To explore whether feature fusion techniques help to enhance the performance of the classification model, we introduced four methods of feature fusion. Radiomics features and deep learning features were fused either by addition or concatenation. The overall training objective incorporated cross-entropy loss ${\mathcal{L}}_{ce}$ to evaluate classification errors, along with contrastive learning loss ${\mathcal{L}}_{cl}$ [23] to enhance the domain invariance of semantic features from pN+ and pN- cases across different centers. To mitigate overfitting, we also trained a momentum model following [24]. This model, which evolves continuously, shares initial parameters with the base model. The parameters of the momentum model are updated via the exponential moving average (EMA).

In the training process, we employed a batch size of 12. Optimization was executed using the Adam optimizer, with an initial learning rate of 10^–4^. The training persisted for a total of 100 epochs, with cosine decay applied to the learning rate starting from the fifth epoch. Our compiling platform was based on the Python library (version 3.7.16) and Pytorch library (version 1.10.0) with CUDA (version 10.2) for GPU (NVIDIA Tesla V100, nvidia corporation, Santa Clara, California, USA) acceleration on a Linux operating system (Ubuntu 16.04 long-term support of a 64-bit server, 40 CPUs, and 503 GB of memory). Our codes are publicly available at https://github.com/paradisetww/OLNM_Prediction.

### Statistical analysis

Statistical analysis was conducted using R software (version 4.0.2). The clinical characteristics across the training, internal validation, and two independent test sets were analyzed using the ${\chi }^{2}$-test for categorical variables and the Kruskal-Wallis H-test for continuous variables to assess distribution differences. The area under the receiver operating characteristic (ROC) curve (AUC), accuracy, sensitivity, specificity, positive predictive value (PPV), and negative predictive value (NPV) were used to evaluate the performance of all models in predicting OLNM of SPILAC patients. The comparison between the ROC curves of different models was conducted using DeLong’s test. A *p*-value of less than 0.05 indicated statistical significance.

## Results

### Patient clinical characteristics

Our study comprised a total of 1325 SPILAC patients. This included 478 pN+ cases and 847 pN- cases. Patient clinical characteristics -- gender, age, nodule maximum diameter, density, and location relative to lung structures -- are detailed in Table 1. Solid nodules show a CTR of 1, while part-solid nodules have a CTR between 0.5 and 1. Comparisons across the training, internal validation, and two independent test sets showed significant inter-cohort differences.

**Table 1:** Clinical characteristics across the training, internal validation, and independent test sets (test set 1 and test set 2) in patients

**Characteristics**	**Training Set**		**Internal Validation Set**		**Independent Test Set 1**		**Independent Test Set 2**		***P*-value**
	**pN+ (*n* = 235)**	**pN- (*n* = 235)**	**pN+ (*n* = 101)**	**pN- (*n* = 101)**	**pN+ (*n* = 43)**	**pN- (*n* = 184)**	**pN+ (*n* = 99)**	**pN- (*n* = 327)**	
Age^a^ (years)	59.9 ± 9.2	59.1 ± 9.9	60.0 ± 9.7	61.7 ± 9.0	59.4 ± 10.6	61.9 ± 9.6	59.7 ± 9.4	59.7 ± 9.7	0.048
Gender									< 0.001
Male	108 (46.0)	101 (43.0)	47 (46.5)	42 (41.6)	21 (48.8)	90 (48.9)	46 (46.5)	146 (44.6)	
Female	127 (54.0)	134 (57.0)	54 (53.5)	59 (58.4)	22 (51.2)	94 (51.1)	53 (53.5)	181 (55.4)	
Maximum Diameter^a^ (cm)	1.8 ± 0.4	1.6 ± 0.4	1.8 ± 0.4	1.6 ± 0.3	1.8 ± 0.4	1.8 ± 0.6	1.8 ± 0.5	1.6 ± 0.4	0.040
Density									< 0.001
Solid	201 (85.5)	198 (84.3)	85 (84.2)	88 (87.1)	39 (90.7)	137 (74.5)	83 (83.8)	239 (73.1)	
Part-Solid	34 (14.5)	37 (15.7)	16 (15.8)	13 (12.9)	4 (9.3)	47 (25.5)	16 (16.2)	88 (26.9)	
Location									< 0.001
Inner	21 (8.9)	11 (4.7)	12 (11.9)	5 (5.0)	8 (18.6)	8 (4.3)	8 (8.1)	17 (5.2)	
Middle	59 (25.1)	54 (23.0)	29 (28.7)	34 (33.7)	9 (20.9)	41 (22.3)	24 (24.2)	106 (32.4)	
Outer	155 (66.0)	170 (72.3)	60 (59.4)	62 (61.4)	26 (60.5)	135 (73.4)	67 (67.7)	204 (62.4)	

Unless otherwise specified, the data presented were the number of patients, with the corresponding percentages denoted in parentheses. The *p*-values represented the outcomes of univariable association analysis conducted for each characteristic across four datasets

*SD* Standard deviation

^a^Data were means ± SDs

### Model performance evaluation

**Fig. 3:**
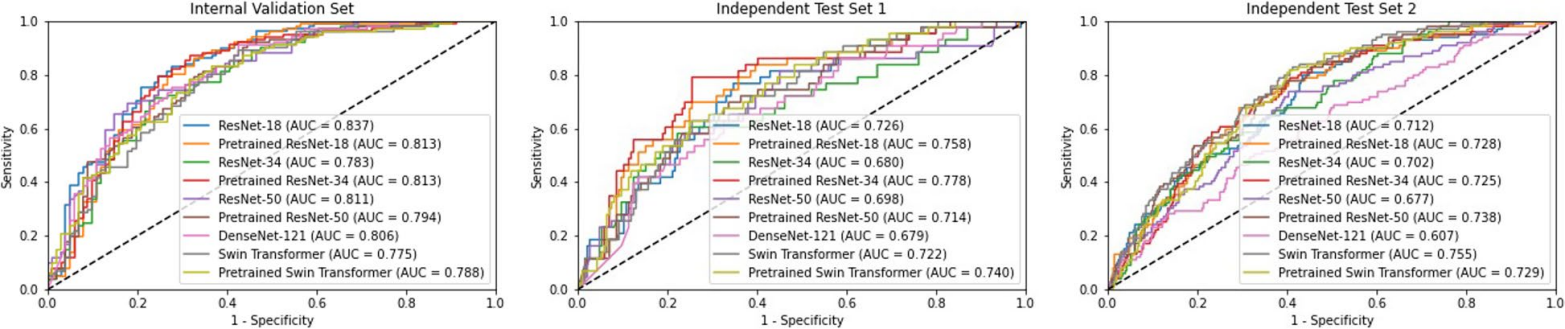
The ROC curves of nine radiomics-deep learning fusion models using concatenation on the internal validation set, independent test set 1, and independent test set 2. The numbers in parenthesis represent the AUC metrics

The accuracy, sensitivity, specificity, PPV, NPV, and AUC of the nine radiomics-deep learning fusion models using concatenation based on ResNet-18, pre-trained ResNet-18, ResNet-34, pre-trained ResNet-34, ResNet-50, pre-trained ResNet-50, DenseNet-121, Swin Transformer, and pre-trained Swin Transformer, on the internal validation set, independent test set 1, and independent test set 2 are shown in Table 2 and Fig. 3. The average AUC (aAUC) of the three datasets trained with ResNet-18, pre-trained ResNet-18, ResNet-34, pre-trained ResNet-34, ResNet-50, pre-trained ResNet-50, DenseNet-121, Swin Transformer, and pre-trained Swin Transformer were 0.746 ± 0.018, 0.754 ± 0.018, 0.710 ± 0.019, 0.746 ± 0.018, 0.722 ± 0.019, 0.733 ± 0.018, 0.674 ± 0.020, 0.742 ± 0.018, 0.737 ± 0.018, respectively. The radiomics-deep learning fusion model trained with pre-trained ResNet-18 obtained the highest aAUC (0.754 ± 0.018), whereas the models trained with ResNet-34 (0.710 ± 0.019) and DenseNet-121 (0.674 ± 0.020) performed the worst. On the internal validation set, there was no significant difference discerned in the AUC of the fusion model, combining radiomics and deep learning, trained with a pre-trained ResNet-18 when compared to other models. However, a distinct performance improvement was noted on the independent test set 1, where the AUC of the radiomics-deep learning fusion model trained with the pre-trained ResNet-18 significantly exceeded those of the models trained with ResNet-34, ResNet-50, pre-trained ResNet-50, DenseNet-121, and Swin Transformer. The respective *p*-values were 0.009, 0.038, 0.022, 0.019, and 0.044, illustrating statistical significance. Similarly, on the independent test set 2, the AUC of the radiomics-deep learning fusion model trained with the pre-trained ResNet-18 demonstrated significantly higher performance than the models trained with ResNet-50 (*p* = 0.028) and DenseNet-121 (*p* < 0.001).

**Table 2:** Comparison of performance among deep learning prediction models integrated with radiomics using concatenation, including ResNet-18, ResNet-34, ResNet-50, DenseNet-121, and Swin Transformer, on the internal validation set, independent test set 1, and independent test set 2

**Model**	**Dataset**	**Accuracy**	**Sensitivity**	**Specificity**	**PPV**	**NPV**	**AUC**	**95% CI**	**aAUC (SD)**
ResNet-18	Internal Validation Set	0.777	0.832	0.723	0.750	0.811	0.837	0.781–0.892	0.746 (0.018)
	Independent Test Set 1	0.674	0.767	0.652	0.340	0.923	0.726	0.644–0.807	
	Independent Test Set 2	0.620	0.798	0.566	0.357	0.902	0.712	0.657–0.768	
ResNet-18^a^	Internal Validation Set	0.767	0.861	0.673	0.725	0.829	0.813	0.753–0.874	0.754 (0.018)
	Independent Test Set 1	0.736	0.698	0.745	0.390	0.913	0.758	0.679–0.838	
	Independent Test Set 2	0.700	0.677	0.706	0.411	0.878	0.728	0.673–0.782	
ResNet-34	Internal Validation Set	0.738	0.713	0.762	0.750	0.726	0.783	0.720–0.847	0.710 (0.019)
	Independent Test Set 1	0.740	0.581	0.777	0.379	0.888	0.680	0.586–0.775	
	Independent Test Set 2	0.636	0.667	0.627	0.351	0.861	0.702	0.647–0.757	
ResNet-34^a^	Internal Validation Set	0.777	0.871	0.683	0.733	0.841	0.813	0.752–0.874	0.746 (0.018)
	Independent Test Set 1	0.753	0.791	0.745	0.420	0.938	0.778	0.699–0.856	
	Independent Test Set 2	0.638	0.788	0.593	0.370	0.902	0.725	0.672–0.778	
ResNet-50	Internal Validation Set	0.757	0.693	0.822	0.795	0.728	0.811	0.752–0.870	0.722 (0.019)
	Independent Test Set 1	0.599	0.814	0.549	0.297	0.927	0.698	0.606–0.790	
	Independent Test Set 2	0.629	0.737	0.596	0.356	0.882	0.677	0.619–0.736	
ResNet-50^a^	Internal Validation Set	0.733	0.911	0.554	0.672	0.862	0.794	0.732–0.856	0.733 (0.018)
	Independent Test Set 1	0.670	0.698	0.663	0.326	0.904	0.714	0.631–0.797	
	Independent Test Set 2	0.660	0.747	0.633	0.381	0.892	0.738	0.687–0.790	
DenseNet-121	Internal Validation Set	0.743	0.911	0.574	0.681	0.866	0.806	0.747–0.866	0.674 (0.020)
	Independent Test Set 1	0.656	0.628	0.663	0.303	0.884	0.679	0.593–0.766	
	Independent Test Set 2	0.540	0.687	0.495	0.292	0.839	0.607	0.543–0.671	
Swin Transformer	Internal Validation Set	0.733	0.891	0.574	0.677	0.841	0.775	0.711–0.840	0.742 (0.018)
	Independent Test Set 1	0.722	0.628	0.745	0.365	0.895	0.722	0.643–0.800	
	Independent Test Set 2	0.643	0.828	0.587	0.378	0.919	0.755	0.705–0.805	
Swin Transformer^a^	Internal Validation Set	0.733	0.832	0.634	0.694	0.790	0.788	0.725–0.850	0.737 (0.018)
	Independent Test Set 1	0.727	0.628	0.750	0.370	0.896	0.740	0.661–0.818	
	Independent Test Set 2	0.646	0.818	0.593	0.379	0.915	0.729	0.677–0.781	

95% CI indicated the 95 percent confidence interval of AUC

^a^Pre-trained models on large-scale medical data

**Fig. 4:**
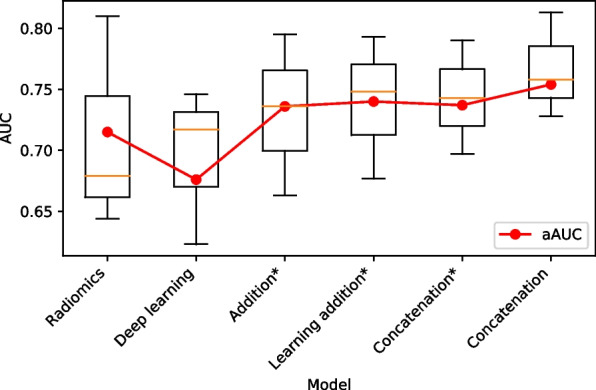
Performance of six models based on radiomics, deep learning, and radiomics-deep learning fusion. Radiomics employed the support vector machine model, while deep learning utilized the pre-trained ResNet-18 model. Addition^*^, learnable addition^*^, concatenation^*^, and concatenation indicate different feature fusion techniques. ^*^ represents that before feature fusion, the radiomics features were mapped to the same dimension as the deep learning features

To further investigate the impact of feature fusion techniques on classification performance, we evaluated six models grounded in radiomics, deep learning, and radiomics-deep learning fusion across three datasets: the internal validation set, independent test set 1, and independent test set 2, as shown in Table 3 and Fig. 4. The aAUC for the three datasets, trained with radiomics, deep learning, and radiomics-deep learning fusion utilizing addition^*^, learnable addition^*^, concatenation^*^, or simple concatenation, were 0.715 ± 0.019, 0.676 ± 0.021, 0.736 ± 0.018, 0.740 ± 0.017, 0.737 ± 0.018, and 0.754 ± 0.018, respectively. Here, the asterisk(^*^) denotes that radiomics features were mapped to the same dimensional space as the deep learning features prior to the feature fusion process. Radiomics leveraged a support vector machine model, whereas deep learning implemented a pre-trained ResNet-18 model. The model achieving the highest aAUC was the radiomics-deep learning fusion model using concatenation (0.754 ± 0.018), while those trained solely with radiomics (0.715 ± 0.019) and deep learning (0.676 ± 0.021) exhibited the least effective performance. Within the internal validation set, the AUC of the radiomics-deep learning fusion model employing concatenation was significantly greater than that of the model trained exclusively with deep learning (*p* = 0.027). Within the independent test set 1, the AUC of the radiomics-deep learning fusion model using concatenation significantly outperformed those of the models trained with radiomics and the radiomics-deep learning fusion utilizing addition^*^, learnable addition^*^, and concatenation^*^, with respective *p*-values of 0.001, 0.002, 0.002, and 0.012. Similarly, within the independent test set 2, the AUC of the radiomics-deep learning fusion model using concatenation was significantly superior to that of the model trained solely with deep learning (*p* < 0.001).

**Table 3:** Performance comparison of different feature fusion techniques on the internal validation set, independent test set 1, and independent test set 2

**Model**	**Feature Fusion**	**Internal Validation Set**	**Independent Test Set 1**	**Independent Test Set 2**	**aAUC (SD)**
Radiomics	None	0.810	0.644	0.679	0.715 (0.019)
Deep Learning	None	0.746	0.717	0.623	0.676 (0.021)
Radiomics-Deep Learning Fusion	Addition^a^	0.795	0.663	0.736	0.736 (0.018)
	Learnable Addition^a^	0.793	0.677	0.748	0.740 (0.017)
	Concatenation^a^	0.790	0.697	0.743	0.737 (0.018)
	Concatenation	0.813	0.758	0.728	0.754 (0.018)

Performance was reported in terms of AUC. Radiomics employed the support vector machine model, while deep learning utilized the pre-trained ResNet-18 model

^a^Before feature fusion, the radiomics features were mapped to the same dimension as the deep learning features

## Discussion

In this study, our primary investigation focused on the efficacy of various factors--model architectures, data initialization strategies, and feature fusion techniques--in enhancing the generalizability to predict OLNM in SPILAC across multiple centers. The experimental outcomes indicated that the integration of the ResNet-18 network architecture, the pre-training strategy, and the concatenation-based fusion technique of radiomics-deep learning features yielded the highest predictive performance (aAUC: 0.754 ± 0.018). In contrast, the models trained from scratch using ResNet-34 or DenseNet121 within a concatenation-based radiomics-deep learning fusion framework, or the models trained separately using radiomics or deep learning, demonstrated inferior performance, with aAUCs of 0.710 ± 0.019, 0.674 ± 0.020, 0.715 ± 0.019, and 0.676 ± 0.021, respectively. These results would be discussed from three perspectives: (1) When compared to other model architectures based on CNN or Transformer, the ResNet-18 outperformed, thanks to its shallower network depth and relatively simplistic structure, which effectively minimized model overfitting. (2) As opposed to training from scratch, employing a pre-training strategy on a large-scale medical dataset followed by fine-tuning on a self-built dataset significantly and universally augmented the model’s generalizability on the independent test set 1 and independent test set 2 obtained from other centers. This improvement can be attributed to the model’s ability to learn diverse and useful feature representations from large-scale medical data, which can be transferred efficaciously to the downstream OLNM prediction task. (3) Radiomics methods provide interpretability, but their scope is limited to the VOIs. Conversely, deep learning methods possess the ability to automatically learn and prioritize task-relevant critical regions. However, the interpretability of deep learning models remains constrained. Compared to models trained separately using radiomics and deep learning, or other radiomics-deep learning feature fusion models, the concatenation-based fusion technique of radiomics-deep learning features effectively combined the strengths of both radiomics and deep learning, thereby achieving superior performance.

The non-invasive preoperative prediction of OLNM status plays a critical role in the treatment decision-making process for patients suffering from SPILAC. However, the robustness and practicality of most existing radiomics or deep learning models for lymph node metastasis prediction were debatable, as they were typically constructed based on limited or single-center datasets [16, 17]. Our research differed in that we collected an extensive array of CT images from six different hospitals, utilizing multiple scan devices and a variety of image reconstruction parameters. The broad distribution of this dataset has enabled us to conduct a comprehensive investigation into the impacts of model architectures, data initialization strategies, and feature fusion techniques on the challenging task of predicting OLNM, bringing our work into closer alignment with real-world clinical scenarios.

Our study does present several limitations. First, despite the comprehensive inclusion of a significant number of multi-center SPILAC patients for OLNM prediction, our study possessed an inherent bias, owing to its retrospective design. Going forward, a prospective exploration would be valuable to mitigate this bias. Second, we have not yet assessed the actual benefits of non-invasive preoperative OLNM prediction for patients with SPILAC. This important area merits further investigation in the future.

## Conclusions

The integration of the ResNet-18 network architecture, the pre-training strategy, and the concatenation-based fusion technique of radiomics-deep learning features have the potential to enhance the generalizability of predicting OLNM based on preoperative CT images in SPILAC across multiple centers.

### Supplementary Information

**Additional file 1: Appendix A.** CT imaging protocols. **Appendix B.** Radiomics model development.

## Data Availability

The data and materials used or analyzed during the study are available upon reasonable request through the corresponding authors.

## Abbreviations

AUC: Area under the curve
CNN: Convolutional neural network
CT: Computed tomography
CTR: Consolidation-to-tumor ratio
EMA: Exponential moving average
GGO: Ground-glass opacity
MR: Magnetic resonance
NPV: Negative predictive value
OLNM: Occult lymph node metastasis
PACS: Picture archiving and communication system
PET: Positron emission tomography
pN-: Pathological nodal negative
pN+: Pathological nodal positive
PPV: Positive predictive value
ROC: Receiver operating characteristic
SPILAC: Solid-predominantly invasive lung adenocarcinoma
VOI: Volume of interest
