# Supplementary material for: Predicting occult lymph node metastasis in solid-predominantly invasive lung adenocarcinoma across multiple centers using radiomics-deep learning fusion model
Source: Cancer Imaging. 2024 Jan 12;24:8. doi: 10.1186/s40644-024-00654-2 (PMC10785418; doi:10.1186/s40644-024-00654-2)
Supplement: Supplementary file 1 — Additional file 1: Appendix A. CT imaging protocols. Appendix B. Radiomics model development. [file 40644_2024_654_MOESM1_ESM.docx]

**Appendix A CT imaging protocols**

This dataset was collected from six hospitals: Shanghai Cancer Center, Fudan University, Shanghai, China (hospital 1), Shanghai Public Health Clinical Center, Fudan University, Shanghai, China (hospital 2), Affiliated Hospital, Nantong University, Nantong, China (hospital 3), Tumor Hospital, Nantong University, Nantong, China (hospital 4), Huadong Hospital, Fudan University, Shanghai, China (hospital 5), and Zhongshan Hospital, Fudan University, Shanghai, China (hospital 6). In the six involved hospitals, CT scans were conducted using one of the following scanners: Siemens Somatom Definition, Siemens Somatom Perspective, Siemens Somatom Emotion 16, Siemens Somatom go.All, Siemens Sensation 64, Toshiba Aquilion one, Philips Brilliance 64, Philips Brilliance iCT 256, UIH uCT 760, UIH uCT 780, UIH uCT 860, UIH uCT 960, GE Medical Systems Revolution CT, GE Optima CT 680 Expert, and Hitachi Medical Corporation Scenaria. The CT scans were reconstructed using one of the following kernels: standard, LUNG, B 30f, B 31f, B 40f, B 50f, FC18, FL 03, Br 40, B_sharp_C. All patients underwent spiral CT scans from the apex to the base of the lung. The scans were performed at tube voltages ranging from approximately 100 to 135 kV, with the tube current set to automatic milliamperes. The reconstructed slice thicknesses were 0.625, 1.000, 1.250, or 1.500 mm.

**Appendix B Radiomics model development**

The 1364 radiomic features could be categorized into seven groups: shape features, first-order features, gray-level co-occurrence matrix (GLCM) features, gray-level run-length matrix (GLRLM) features, gray-level size zone matrix (GLSZM) features, gray-level dependence matrix (GLDM) features, and neighborhood gray-tone difference matrix (NGTDM) features. These radiomic features were extracted from the original image and four derived images obtained using filters, including box sigma image, Laplacian of Gaussian (LoG) image, wavelet transformed image, and Laplacian sharpening image. The box sigma image was generated by applying a box filter to the image for smoothing, reducing noise interference. The LoG image was obtained by utilizing LoG filters with different kernel sizes, specifically 0.5, 1, 2, and 4 in this study. Wavelet transformation was performed in three dimensions using low (L) or high (H) pass filters, resulting in eight decomposed images: LLL, LLH, LHL, LHH, HLL, HLH, HHL, and HHH. Laplacian sharpening highlighted the high-frequency components of the image using the Laplacian operator, enhancing edges and details. **Table B1** presents all types of extracted radiomic features.

**Table B1** List of categories for all extracted radiomics features

| **Category** | **Original Image** | **Box Sigma** | **Laplacian of Gaussian** | **Wavelet Transformation** | **Laplacian Sharpening** | **All** |
| --- | --- | --- | --- | --- | --- | --- |
| Shape | 14 |  |  |  |  | 14 |
| First-Order | 18 | 18 | 72 | 144 | 18 | 270 |
| GLCM | 21 | 21 | 84 | 168 | 21 | 315 |
| GLRLM | 16 | 16 | 64 | 128 | 16 | 240 |
| GLSZM | 16 | 16 | 64 | 128 | 16 | 240 |
| GLDM | 14 | 14 | 56 | 112 | 14 | 210 |
| NGTDM | 5 | 5 | 20 | 40 | 5 | 75 |
| Total | 104 | 90 | 360 | 720 | 90 | 1364 |

The top ten radiomic features selected through the decision tree were as follows:

- boxsigmaimage_firstorder_Minimum
- wavelet_glrlm_wavelet.HHH.ShortRunEmphasis
- wavelet_glcm_wavelet.LHH.InverseVariance
- boxsigmaimage_firstorder_10Percentile
- wavelet_glszm_wavelet.HHL.LargeAreaLowGrayLevelEmphasis
- boxsigmaimage_glrlm_LongRunLowGrayLevelEmphasis
- laplaciansharpening_firstorder_90Percentile
- wavelet_gldm_wavelet.LHL.DependenceVariance
- log_glcm_log.sigma.0.5.mm.3D.Imc1
- wavelet_glcm_wavelet.LLL.MaximumProbability

Based on these ten radiomic features, a prediction model for OLNM was constructed using the support vector machine.
